# Landmarks

**Published:** 1893-06

**Authors:** W. H. Whitslar


### Landmarks.

President's Address, Northern Ohio Dental Association, Akron, O., May 9th, 1893

BY W. H. WHITSLAR, M.D., D.D.S.

*Gentlemen of the Northern Ohio Dental Association:* This meeting is the thirty-fourth annual landmark of this Association. We look forward to these landmarks because of the bond of professional and social union which the members of this society enjoy. There are reasons for this affection, and, among the first is its age. Born November 3d, 1857, at Tremont Hall, Cleveland, O., the child began to grow, and now one hundred and thirty active members have their names upon the roll. This meeting should be called the thirty-sixth landmark, but, as the charter was not obtained until 1859, we have numbered these meetings from that time. The Northern Ohio Dental Association was the second society formed in this state, at least, that had any permanency. The Mississippi Valley society was the first. Like a number of these societies which were of early formation, the Northern Ohio, has in its progress, made itself felt in the history of the dental profession by means of some of its members attaining world-wide reputation. In this society, years ago, that seer whose knowledge and skill were sufficient to entitle him to the name of the "Father of Dentistry," Dr. William H. Atkinson, made his debut in dental society. He was a notable example of generosity and professional ability. Others, such as Doctors B. Strikland, B. F. Robinson, F. S. Sosson, H. H. Newton, and John Stephan, who have passed out of this world and now have their abode in the Eternal City, and have left good works and tender memories behind them, which we as a society, individually and collectively, must revere.

Among the living actively honoring our profession are the names of Charles R. Butler, Corydon Palmer, J. A. Robison, of Jackson, Mich., and a host of others whose names are familiar to the readers of our Journals. These, and our honorary members, have by their presence and assistance given this society an impetus, which, like Tennyson's "Brook," goes on forever.

We have to feel then that this society is one of the landmarks among the one hundred and thirty societies of the country.

The present year, 1893, with its three great dental congressess, marks an epoch in dentistry. These are the World's Columbian Dental Congress at Chicago, The International Medical Congress at Rome, Italy, and the Pan American Medical Congress at Washington, D. C.

The last two whilst bearing the name of Medical Congress, have Sections devoted to dental and oral surgery. Such recognition of dentistry as a specialty of medicine is gratifying.

The birth of this series of landmarks occurred when the American Medical Association adopted the resolution presented by Nathan S. Davis M.D., of Chicago, recognizing dental science as a specialty of medicine. The resolution, however, is specific in its demand that the medical studies in the dental schools should be equal to those of the medical schools.

The Columbian Dental Congress at Chicago, in August, will be a glorious event. Our efforts will be reviewed by foreign critics and the sovereignty of "American Dentistry," if such a term is permissible, must be maintained. It is the duty of every reputable dentist to aid the management in making the Congress a grand success, and an indisputable landmark for all ages.

Dentistry is not a new vocation. Egyptians were the first dentists that we learn of. Etrurians practiced dentistry as a specialty of medicine, and bridgework was performed by these people twenty-four hundred years ago.

Erasistratus extracted teeth with forceps 2000 B. C. Esculapius 1250 B. C. used a narcotic to produce insensibility for tooth extraction.

Celsus introduced gold foil for filling teeth 14 A. D. During the reign of Louis XIV in France and George II in England, royal authority proclaimed dentistry a profession distinct and separate from the art of shaving and hair dressing.

This might be called the first landmark of dentistry as a profession, and also its distinction from a trade.

John Woofendale was the first dentist in America. Of the Educational landmarks, Chapin A. Harris organized the first dental college. Now thirty-eight colleges dot our country as educational centers. Correlated to these are the National Association of Faculties and the National Association of Dental Examiners. These bodies enhance the work of the colleges.

The American, Southern, and Women's Dental Associations, and the Post-graduate Reading Courses, together with the various state associations are all landmarks of importance. The first dental journal published was the American Journal and Library of Dental Science. The Journals of the present year are vastly superior to what they have been in former years. Our Ohio Journal as well as the Cosmos and the Items of Interest have made great strides in journalism.

Diversified topics have been chosen as subjects for text-books during past five years. A remarkable scarcity of suitable text-books for students previously existed. The "American System of Dentistry" is a work almost worthy of adoption as a text-book in toto for students. But, it is not complete without "Black's Dental Anatomy," and, "Miller's Micro-organisms of the Mouth" (complete), both decided landmarks in the progress of dental publication. Every intelligent dentist would do well to become a master of these works. Farrar's also Talbot's works in "Irregularities of the teeth," "Mitchell's Chemistry," "Rehfuss' Dental Jurisprudence," "Evans' Bridgework," Garretson's Oral Surgery," "Kingsley's Oral Deformities," are all modern works.

From a medical standpoint perhaps the greatest landmark the dental profession can offer is that of the discovery that nitrous oxide gas produces anæsthesia.

In practical experience we must acknowledge landmarks in the discovery of the rubber dam, dental engine, cohesive gold amalgam, cement, mallets, uses of electricity, and not the least among these, porcelain teeth ; all aid in giving serviceable aid to our patients.

In the State of Illinois, December 4th, 1888, one of the greatest landmarks of the dental profession was enacted. I refer to the incoporation of the Dental Protective Association. We cannot honor Dr. J. N. Crouse, the founder of this association too much, for, by this protective agency, the profession has saved more than a million of dollars per year from patent companies. It is a duty every dentist owes himself, family, and profession to become a member of the Dental Protective Association.

In concluding let me say that I intended to be brief in this address. Let us congratulate ourselves upon the fact that our society is prosperous. One of the reasons for this prosperity is that the business of the society is conducted by the Executive Committee.

Unlike most societies we can boast of plenty of money in the treasury, in thirty-six years having had but three treasurers, and, no yearly dues are exacted from its members.

